# Three-dimensional multi-physics modelling and optimisation of a hybrid of radiation filtering and passive cooling strategy for floating photovoltaic systems

**DOI:** 10.1038/s41598-025-10409-z

**Published:** 2025-07-25

**Authors:** Bayu Sutanto, Hector Iacovides, Adel Nasser, Andrea Cioncolini, Imran Afgan

**Affiliations:** 1 Thermo-Fluids Group, Department of Mechanical and Aerospace Engineering, University of Manchester, Manchester, M13 9PL UK; 2 Department of Mechanical Engineering, Politeknik Negeri Semarang, Jl. Prof. H. Soedarto S.H., Tembalang, Semarang 50275 Indonesia; 3 Department of Mechanical Engineering (Robotics), Guangdong Technion - Israel Institute of Technology, 241 Daxue Road, Shantou, 515063 Guangdong China; 4 Department of Mechanical and Nuclear Engineering, Khalifa University of Science and Technology, P.O. Box 127788, Abu Dhabi, United Arab Emirates

**Keywords:** Floating photovoltaic, Multi-physics modelling, Natural convection, Radiation filter, Solar energy, Photovoltaics

## Abstract

This study presents the development of a three-dimensional multi-physics thermal model for a novel design of a floating photovoltaic system, which incorporates a natural convection cooling loop where the coolant also acts as solar radiation filter. The thermal model is employed to investigate the combined effects of the passive cooling loop and the radiation filtering of the cooling channel located above the photovoltaic module, by varying the height of the cooling channel and the spacing between connecting tubes, with a view at optimising the cooling loop geometry. Simulation results indicate that the combination of radiation filtering and passive cooling is remarkably effective at improving the electrical performance of the floating photovoltaic system, by filtering out, from solar radiation, the non-useful range of wavelengths for crystalline silicon photovoltaic cells and, at the same time, reducing operational temperatures. Specifically, the daily average temperature of the floating photovoltaic system can be reduced by up to 15 °C, leading to a 3% increase in electrical efficiency and a contribution in thermal convective cooling by up to 64.48%. Additionally, the system’s performance is assessed under varying global solar irradiance levels, representing possible maximum and minimum conditions worldwide at any time of the year. The results demonstrate the practical feasibility of the proposed design, which can reduce the operating temperature of the solar panels even under minimum irradiance levels.

## Introduction

A floating photovoltaic (FPV) system, comprising solar panels that are mounted atop of a floating structure and deployed over water bodies, is an innovative solution to tackle the central problem of extensive and expensive land requirements for solar farms^1^. Additional advantages of FPV systems are the reduction of the rate of evaporation from the water body, and the possibility of using the latter as a heat sink for the thermal management of the solar panels^2^. It is well known that the operating temperature is one of the key parameters in the efficiency of solar panels^3^, because increasing the temperature of photovoltaic (PV) cells reduces the power output^4^ and shortens the length of their operating life^5^. For every 1 degree Celsius rise in temperature of a crystalline silicon (c-Si) PV cell, the electrical efficiency is decreased by around half a percentage point on average^6^. Consequently, effective thermal management techniques can play a vital role in sustaining and improving energy output of PV system^7^.

In this context, photovoltaic/thermal (PV/T) systems—hybrid collectors that simultaneously generate electrical and thermal energy—have gained increasing attention^8^. Experimental investigations have shown that active water cooling in PV/T systems can significantly improve performance by lowering PV cell temperatures and increasing total energy output. Specifically, circulating water through the system can reduce PV cell temperatures by over 10 °C, leading to an approximate 6% increase in electricity production^9^. This enhanced cooling effect not only stabilises PV cell temperatures but also allows recovery of useful thermal energy, making water-cooled PV/T systems markedly more efficient than standalone PV modules^10,11^.

These findings align with thermal management strategies applied in FPV systems. The most common thermal management methodologies presently employed with FPV are water veil cooling^12,13^ and internally pumped water cooling^14^. The use of water veil cooling was reported to reduce the FPV temperature from 45 °C to 28 °C with a water pumping flow rate of 2 L/min, and the yearly energy gain was between 8–12%^13^. Regarding the internally pumped water cooling methodology^14^, available evidence indicates that using a water flow rate of 13 L/min can reduce the FPV temperature within the range of 19.9 to 33.3 °C , with a corresponding increase in electrical power output of 7.4‒13.2%, depending on solar irradiance levels between 319.5 and 901.9 W/m^2^. These cooling systems, however, need pumping energy to circulate cooling water. Consequently, our recent research efforts^15,16^ have been focused on the use of a natural convection cooling loop (NCCL) system, which is entirely passive and does not require energy supply to operate. We demonstrated that under solar irradiance ranging from 350 to 1050 W/m^2^ the NCCL system, without the use of any pumping power, can reduce operational temperature by up to 3.8 °C on average^16^.

Given these promising results, further development of the NCCL system is needed to optimise the cooling design for FPV applications. Collective studies highlight the potential of improving FPV performance by reducing temperature and filtering solar irradiance to benefit silicon PV cells with a 1.12 eV band gap^17^. The electrical performance of c-Si PV cells is influenced by temperature and the material’s band gap energy^18^. Therefore, the NCCL system can be extended to not only reduce FPV temperature but also to filter out photons that do not fall within the required band gap energy for c-Si PV cells. The band gap energy, 1.12 eV for silicon, represents the minimum photon energy needed to excite electrons in the PV cell, allowing silicon to extract electrical energy from wavelengths between 325 and 1125 nm^19^. Photons outside this range cannot be absorbed and are wasted as heat instead of being converted into electricity^20^. Since most solar irradiance spans wavelengths from 250 to 2500 nm, not all can be utilised by c-Si PV cells. Installing a radiation filter in front of the PV panel is the natural solution to control the incident solar spectrum^21^. Notably, pure water can serve as an effective radiation filter because it has low transmissivity beyond 1500 nm, absorbing longer wavelengths (low-energy photons)^22^. Therefore, in this study we introduce a novel approach that incorporates radiation filtering within the NCCL system for FPV panels. Basically, in the proposed system the cooling channel is realised in front of the PV panel, so that the flowing water can simultaneously act as solar radiation filter and as coolant. The proposed system aims to reduce PV cell temperature and absorb non-useful wavelengths before they reach the c-Si PV cell. To our knowledge, research has yet to be conducted using this method.

Having in our previous research efforts developed two-dimensional simulation models^23,24^, the main objective of this study is to develop a reliable three-dimensional simulation tool to model the buoyancy-driven motion of NCCL for FPV panels. Thermally coupled to solar irradiance interactions through the cooling fluid and PV layers, the resulting simulations will:

- Advance our understanding of the multi-physics interactions in NCCL and radiation filter modelling,
- Optimise the cooling loop geometry in both two- and three-dimensional models,
- Evaluate the system’s performance under various global solar irradiance patterns over a 24-h simulation period at different geographic locations.

## Methods

### Physical model design

**Fig. 1:**
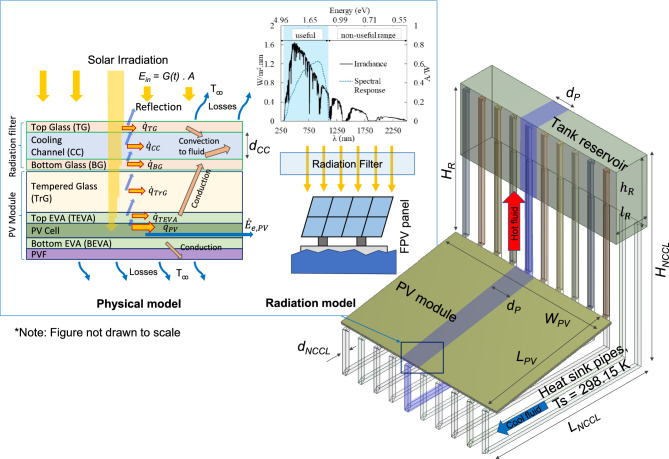
The schematic design of hybrid of radiation filter and NCCL for FPV systems.

Figure 1 shows the schematic design of a hybrid radiation filter and NCCL system for FPV panels, improved from earlier studies^23,24^. The cooling loop consists of three main components: the rectangular cooling section (0.75 m in length (${L}_{PV}$), 1.00 m in width (${W}_{PV}$) and at a tilt angle ($\theta$) of 10° sitting above the PV module, receiving solar energy, which is transmitted to the panel, and removing thermal energy through heat convection from the top surface of the panel; a heat sink section at the base, immersed in water, which removes thermal energy from the cooling loop and rejects it to the body of water; and a reservoir tank at the top.

The NCCL dimensions have been optimised, for two-dimensional conditions^24^, with a horizontal length (${L}_{NCCL}$) of 1.0 m and a vertical height (${H}_{NCCL}$) of 1.2 m, connected by tubes of 20 mm diameter (${d}_{NCCL}$). The top tank reservoir, measuring 0.2 m in length (${l}_{R}$) and 0.4 m in height (${h}_{R}$), is positioned 0.75 m above the PV module (${H}_{R}$).

As already noted, the cooling channel also acts as a radiation filter, absorbing radiation energy over a specific range of wavelengths before the solar irradiance reaches the PV module. As a c-Si PV cell can only convert a specific range of “useful” wavelengths (325 nm – 1125 nm) of solar irradiance into electrical energy, energy outside this range becomes thermal energy (increasing the PV cell temperature^25^). Pure water, which strongly absorbs thermal radiation over the red-infrared region (above 1125 nm), which has very little overlap with the “useful” c-Si PV cell region, can consequently be an effective radiation filter^22^. In this study, pure water is used as a cooling fluid with five different cooling channel heights (${d}_{CC}$) of 1, 2, 5, 10, and 20 mm. The irradiance then arrives at the standard c-Si PV module, consisting of tempered glass, ethylene–vinyl acetate (EVA) layers, a silicon PV cell, and a polyvinyl fluoride (PVF) layer.

The coolant absorbs thermal energy from the lower surface of NCCL and heats up, becoming buoyant and moving to the top tank reservoir above the PV module, where it replaces cooler fluid, pushing it down through the sink and then back to the PV cooling section. This circulation removes thermal energy from the PV panel and, through the heat sink, dissipates it to the body of water.

**Fig. 2:**
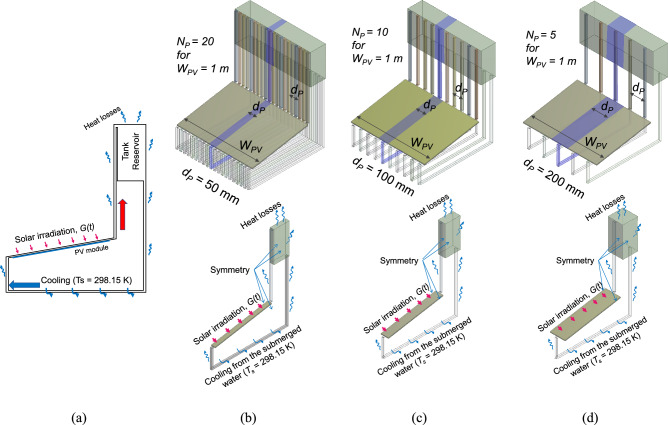
The design and boundary conditions of 2-D model and slice samples of 3-D models with tube spacing, ${d}_{P}$ variations: (**a**) 2-D model, (**b**) 3-D with ${d}_{P}$ = 50 mm, (**c**) 3-D with ${d}_{P}$ = 100 mm, (**d**) 3-D with ${d}_{P}$ = 200 mm.

To minimise the numerical work, a slice of the 3-D model with a width ${d}_{P}$, the tube spacing, is taken from the full width of the NCCL (${W}_{PV}$), as shown in Fig. 2, with symmetry boundary conditions in the spanwise direction. Various tube spacings, ${d}_{P,}$ values (50 mm, 100 mm, and 200 mm) are considered, to study the effect of the number of connecting tubes (${N}_{P}$) and to approximate the ideal 2-D model. For a constant ${W}_{PV}$ of 1 m, these ${d}_{P}$ values result in 20, 10, and 5 connecting tubes, respectively. The 2-D model corresponds to the limiting case of a 3-D model with continuous, unseparated connecting tubes (${d}_{P}$ = 0 mm).

### Thermal modelling

The governing equations of mass, momentum, and energy transport are presented in Eqs. (1)–(3) for the transient model in the closed cooling loop system. Here, ${u}_{i}$ is the velocity vector, and $P$, $T$*,* $\rho$ $,\mu$, $Pr$, $\dot{q}$, and ${c}_{p}$ represent the fluid pressure, temperature, density, dynamic viscosity, Prandtl number, heat rate generation per unit volume, and specific heat capacity, respectively^16^. The source term $\dot{q}$, is included in the energy equation to account for heat generation within each PV module layer due to radiation absorption, and in the case of the PV layer also for electrical power generation, as detailed in "Radiation modelling".

1 $$\frac{\partial \rho }{\partial t}+\frac{\partial \rho {u}_{i}}{\partial {x}_{i}}=0$$

2 $$\frac{\partial }{\partial t}\left(\rho {u}_{i}\right)+\frac{\partial }{\partial {x}_{j}}\left(\rho {u}_{i}{u}_{j}\right)=-\frac{\partial P}{\partial {x}_{i}}+\frac{\partial }{\partial {x}_{j}}\left(\mu \left(\frac{\partial {u}_{i}}{\partial {x}_{j}}+\frac{\partial {u}_{j}}{\partial {x}_{i}}\right)\right)+{\rho g}_{i}$$

3 $$\frac{\partial }{\partial t}\left(\rho T\right)+\frac{\partial }{\partial {x}_{j}}\left(\rho {u}_{j}T\right)=\frac{\partial }{\partial {x}_{j}}\left(\frac{\mu }{Pr}\frac{\partial T}{\partial {x}_{j}}\right)+\frac{\dot{q}}{{c}_{p}}$$

The Boussinesq approximation is used to simplify the buoyancy force in the momentum equations without using temperature-dependent fluid properties, as shown in Eq. (4)^26^. Where $\beta$ is the coefficient of thermal volumetric expansion, defined in Eq. (5). The reference temperature (${T}_{ref}$) is the temperature of the heat sink environment (lake, river, or dam), and ${\rho }_{ref}$ is the density at the reference temperature^16^. Given the limited temperature variation range ($\beta \left(T-{T}_{ref}\right)\ll 1$), the Boussinesq approximation is appropriate for this study.

4 $$\rho \overrightarrow{g}={\rho }_{ref}\overrightarrow{g}\left(1-\beta \left(T-{T}_{ref}\right)\right)$$

5 $$\beta =-\frac{1}{\rho }{\left(\frac{d\rho }{dT}\right)}_{P}$$

The properties of the PV module and the materials involved in this study, such as density ($\rho$), specific heat capacity (${c}_{p}$), and thermal conductivity ($k$) for the natural convection loop, are listed in Table 1.

**Table 1:** The properties of PV modules and materials for NCCL systems, adapted from our previous study^15,24^.

Materials	$\rho$(kg/m^3^)	${c}_{p}$(J/kg.K)	$k$(W/m.K)
Glass	2450	790	0.7
EVA	960	2090	0.311
PV cell	2330	677	130
PVF	1200	1250	0.15

### Radiation modelling

As radiation reaches each layer, a portion of the energy is absorbed (generating thermal energy), whilst some of it is reflected back, and the rest is transmitted based on the layer’s absorptivity, reflectivity, and transmissivity ($\alpha$, $r$, and $\tau$)^27^. Given the known radiation properties of each layer, the rate of thermal energy generation per unit volume ($\dot{q}$) can be calculated by applying the energy balance (Eq. 3) to each layer of the radiation filter box and PV module^24^. Equations (6)‒(11) define the source term ($\dot{q}$) for each layer, where ${\dot{E}}_{\alpha }$*, *${G}_{s}$*,* $V$, $d$, and $A$ denote the absorbed heat energy, incoming solar irradiation, volume, height, and surface area of each layer, respectively. The subscripts $TG$, $CC$, $BG$, $TrG$, $EVA$ and $PV$ refer to the top glass, cooling channel, bottom glass, tempered glass, top EVA, and PV cell layers, respectively. The details of these equations are available in our previous study^24^.

6 $${\dot{q}}_{TG}=\frac{ {\dot{E}}_{\alpha ,TG}}{{V}_{TG}}=\frac{ 1}{{d}_{TG}}{\int }_{0}^{\infty }{\alpha }_{TG}\left(\lambda \right){G}_{s,\lambda }\left(\lambda \right)d\lambda$$

7 $${\dot{q}}_{CC}=\frac{ {\dot{E}}_{\alpha ,CC}}{{V}_{CC}}=\frac{ 1}{{d}_{CC}}{\int }_{0}^{\infty } {\alpha }_{CC}\left(\lambda \right){\tau }_{TG}\left(\lambda \right){G}_{s,\lambda }\left(\lambda \right)d\lambda$$

8 $${\dot{q}}_{BG}=\frac{ {\dot{E}}_{\alpha ,BG}}{{V}_{BG}}=\frac{ 1}{{d}_{BG}}{\int }_{0}^{\infty } {\alpha }_{BG}\left(\lambda \right){\tau }_{CC}\left(\lambda \right){\tau }_{TG}\left(\lambda \right){G}_{s,\lambda }\left(\lambda \right)d\lambda$$

9 $${\dot{q}}_{TrG}=\frac{ {\dot{E}}_{\alpha ,TrG}}{{V}_{TrG}}=\frac{ 1}{{d}_{TrG}}{\int }_{0}^{\infty } {\alpha }_{TrG}\left(\lambda \right){\tau }_{BG}\left(\lambda \right){\tau }_{CC}\left(\lambda \right){\tau }_{TG}\left(\lambda \right){G}_{s,\lambda }\left(\lambda \right)d\lambda$$

10 $${\dot{q}}_{EVA}=\frac{ {\dot{E}}_{\alpha ,EVA}}{{V}_{EVA}}=\frac{ 1}{{d}_{EVA}}{\int }_{0}^{\infty } {\alpha }_{EVA}\left(\lambda \right){\tau }_{TrG}\left(\lambda \right){\tau }_{BG}\left(\lambda \right){\tau }_{CC}\left(\lambda \right){\tau }_{TG}\left(\lambda \right){G}_{s,\lambda }\left(\lambda \right)d\lambda$$

11 $${\dot{q}}_{PV}=\frac{{\dot{E}}_{th,PV}}{{V}_{PV}}=\frac{{\dot{E}}_{\alpha ,PV}-{\dot{E}}_{e,PV}}{{V}_{PV}}=\frac{ 1}{{d}_{PV}}{\int }_{0}^{\infty } {\alpha }_{PV}\left(\lambda \right){\tau }_{EVA}\left(\lambda \right){\tau }_{TrG}\left(\lambda \right){\tau }_{BG}\left(\lambda \right){\tau }_{CC}\left(\lambda \right){\tau }_{TG}\left(\lambda \right){G}_{s,\lambda }\left(\lambda \right)d\lambda -\frac{{\dot{E}}_{e,PV}}{{V}_{PV}}$$

The term ${\dot{E}}_{e,PV}$ in Eq. (11) representing electrical power generation within the PV cell layer, is given by Eq. (12)^28^. Here, ${SR}_{PV}$ is the spectral response of c-Si PV cells as a function of wavelength ($\lambda$). The ${FF}_{ref}$ and ${V}_{ref}$ denote the fill factor and open-circuit voltage at the reference temperature (${T}_{ref}$), while ${C}_{FF}$ and ${C}_{V}$ are the temperature constants for the fill factor and open-circuit voltage.

12 $${\dot{E}}_{e,PV}=\left({FF}_{ref}+{C}_{FF}\left(T-{T}_{ref}\right)\right).\left({V}_{ref}+{C}_{V}\left(T-{T}_{ref}\right)\right).{\int }_{0}^{\infty }{SR}_{PV}\left(\lambda \right){{\tau }_{EVA}\left(\lambda \right)\tau }_{TrG}\left(\lambda \right){{\tau }_{BG}\left(\lambda \right)\tau }_{CF}\left(\lambda \right){\tau }_{TG}\left(\lambda \right){G}_{s,\lambda }\left(\lambda \right).Ad\lambda$$

The temporal variation of global solar irradiance (${G}_{S}$) received by the system is modelled based on the assumption of a clear day^29^, as shown in Eq. (13). Here, ${G}_{D}$ represents the direct solar irradiance, and $AM$ is the air mass.

13 $${G}_{S}=1.1{G}_{D}=1.1\times 1.353\times {0.7}^{{AM}^{0.678}}$$

Equation (14) defines air mass, where ${\theta }_{z}$ is the zenith angle^30^.

14 $$AM=\frac{1}{cos{\theta }_{z}}$$

The zenith angle (${\theta }_{z}$) can be calculated using Eq. (15),

15 $$cos{\theta }_{z}=cos\phi .cos\delta .cos\omega +sin\phi .sin\delta$$

where $\phi$ is the latitude of the location on Earth, $\delta$ is the declination angle (approximated in Eq. (16) for any given day ($n$) of the year), and $\omega$ is the hour angle (defined in Eq. (17), with $LST$ being the local solar time)^31^.

16 $$\delta =23.45sin\left(360\frac{284+n}{365}\right)$$

17 $$\omega =15^\circ (LST-12)$$

This study evaluates various global reservoir locations to determine daily ${G}_{S}$ levels. Table 2 summarises five selected global sites from the northern to the southern hemispheres for FPV power plants. These locations have been chosen based on their potential for FPV deployment and their likelihood of experiencing maximum and minimum solar irradiance due to the sun’s movement across the Earth^32^.

**Table 2:** Sampling locations for global FPV power plants.

Location	Latitude, $\phi$ (degree)	Longitude (degree)	Current installed capacity (MWp)	Water surface area (± m^2^)
Cirata, Indonesia	− 6.69	107.34	145	62.000.000
Warrnambool, Australia	− 38.37	142.51	0.5	10.000
Canoe Brook, USA	40.75	− 74.35	8.9	3.000.000
Dingzhuang, China	37.33	116.43	320	200.000
Franschhoek, South Africa	-33.92	19.06	0.06	10.000

**Fig. 3:**
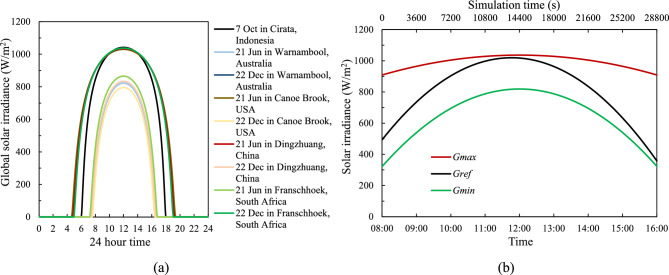
Solar irradiation pattern under clear sky conditions at (**a**) various global locations and (**b**) selected possible maximum and minimum radiation pattern.

Using Eqs. (13)–(15), Fig. 3(a) illustrates global daily solar irradiance patterns from summer to winter, highlighting the potential maximum and minimum ${G}_{S}$ values. To streamline the analysis, Fig. 3(b) focuses on three specific solar irradiance scenarios: maximum possible radiation (${G}_{max}$) on June 21st at Dingzhuang, China (summer solstice), minimum possible radiation (${G}_{min}$) on December 22nd at Canoe Brook, USA (winter solstice), and a reference radiation (${G}_{ref}$), previously established and validated in earlier studies^16,24^. The inclusion of ${G}_{ref}$ serves not only as a consistent benchmark across simulations but also enables verification of the model’s accuracy, as this case has been experimentally validated.

Equation (18) defines these ${G}_{S}$ as a function of time (t) in seconds, starting from 8:00 AM to 4:00 PM ($0\le t\le 28800 s$), with the constants provided in Table 3.

**Table 3:** Constants of the ${G}_{s}$ function of time from 8 am to 4 pm.

Constant	$a\left( {\frac{W}{{m^{2} s^{2} }}} \right)$	$b\left( {\frac{W}{{m^{2} s}}} \right)$	$c\left( {\frac{W}{{m^{2} }}} \right)$
${G}_{max}(t)$	− 6.153 × 10^–7^	1.772 × 10^–2^	909.1
${G}_{ref}(t)$^24^	− 2.854 × 10^–6^	7.747 × 10^–2^	493.4
${G}_{min}(t)$	− 2.397 × 10^–6^	6.905 × 10^–2^	321.9

18 $${G}_{S}(t)=a{t}^{2}+bt+c$$

### Numerical aspects

A conjugate heat transfer analysis is performed to account for the heat conduction across PV module layers and the heat convection within the fluid loop. The time-dependent governing Eqs. (1)–(3) are solved numerically using ANSYS-Fluent, utilising finite-volume discretisation and the semi-implicit method for the pressure-linked equation (SIMPLE) algorithm for pressure calculations^16,24^. The body force-weighted scheme interpolates pressure values, while a second-order upwind scheme discretises convective transport. The transient term is handled with a second-order implicit time discretisation scheme. Convergence is achieved when continuity and velocity residuals fall below 10^–3^ and energy residual below 10^–6^.

### Boundary conditions

In the heat sink section, the inner surface of the pipe is maintained at 298.15 K in the heat sink pipes section to simulate standard test conditions (STC). Heat losses to the environment are calculated using Newton’s law of cooling (Eq. (19)), with the convective heat transfer coefficient ($h$) derived from the Nusselt number ($Nu$) correlations (Eq. (20)). The outside air ambient temperature (${T}_{f}$) is assumed constant at 298.15 K during noon. At night, it is taken to be 9 °C lower than the daytime value, in accordance with typical diurnal temperature variations^33–35^. Additionally, ${T}_{w}$ signifies the wall temperature and $Ra$ and $Pr$ are Rayleigh and Prandtl numbers.

19 $$q"=h({T}_{w}-{T}_{f})$$

20 $$Nu\equiv \frac{hL}{k} ; Nu = f\left(Ra, Pr\right)$$

Free convection releases heat from the outer solid layers, with relevant Nusselt number correlations provided. Equations (21)–(23) give average $Nu$ correlation for free convection from the upper surface, the lower surface of a heated horizontal plate, and hot vertical plates, respectively. The $Nu$ correlation for hot vertical plates in Eq. (23) has constants $C$ and $n$ being 0.59 and 0.25, respectively for laminar flow^36^.

21 $${\overline{Nu} }_{L}= 0.15{Ra}_{L}^{1/3}$$

22 $${\overline{Nu} }_{L}= 0.52{Ra}_{L}^{1/5}$$

23 $${\overline{Nu} }_{L}= C{Ra}_{L}^{n}$$

Equation (24) calculates radiation heat loss from the outer solid surfaces. The average emissivity for glass and PVF is 0.9, and the surrounding sky temperature (${T}_{surr}$) is estimated to be 20 degrees below ambient^37^.

24 $$q"=\varepsilon \sigma ({T}_{w}^{4}-{T}_{surr}^{4})$$

For the initial condition ($t=0s$), the temperature is set to 298.15 K, and the velocity field is zero across the domain.

### Grid independence

A mesh-independence study was conducted by varying the number of control volumes in the solid and fluid domains of a medium cooling channel (${d}_{CC}$ = 5 mm) and medium tube spacing (${d}_{P}$ = 100 mm) under ${G}_{ref}$ irradiation. Four mesh topologies were tested with 496,000; 1,334,000; 3,356,000; 7,136,750; and 14,864,000 control volumes. In this study mesh quality has been maintained, with average skewness at 0.0569 and maximum at 0.57759, while orthogonal quality averaged 0.9789 with a minimum of 0.6165, indicating all meshes were suitable for simulation.

**Fig. 4:**
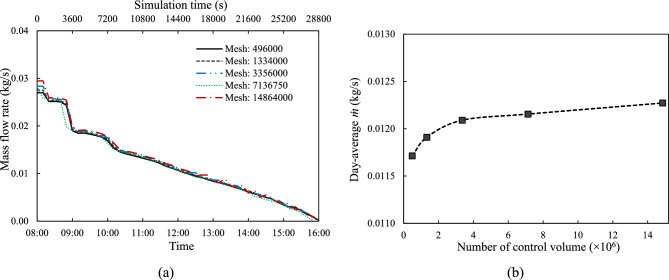
Analysis of $\dot{m}$: (**a**) instantaneous $\dot{m}$ over time, and (**b**) day-averaged $\dot{m}$ changes with different mesh size, for ${d}_{CC}$ = 5 mm and ${d}_{P}$ = 100 mm.

The mass flow rate ($\dot{m}$) is calculated through Eq. (25) considering non-uniform velocity across the cross-sectional area. Fig. 4(a) shows the variation of $\dot{m}$ with time, for the different mesh sizes, and Fig. 4(b) presents the variation of the day-average $\dot{m}$ with mesh size. Initially, $\dot{m}$ increases with more control volumes but stabilises after 3,356,000. This indicates that further mesh refinement yields diminishing returns, validating the mesh independence of the solution at higher resolutions.

25 $$\dot{m}= \int \rho \overrightarrow{V}.d\overrightarrow{A}$$

**Fig. 5:**
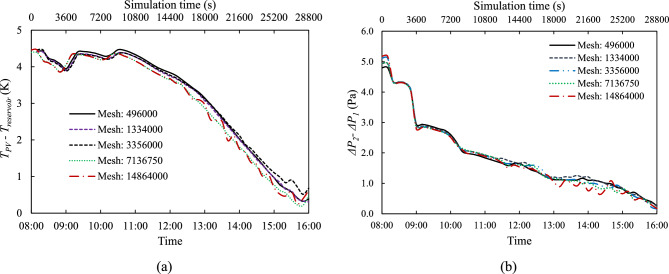
Instantaneous (**a**) ${T}_{PV}-{T}_{reservoir}$ and (**b**) ${\Delta P}_{2}-{\Delta P}_{1}$ over time, for ${d}_{CC}$ = 5 mm and ${d}_{P}$ = 100 mm.

The coolant mass flow rate is driven by the temperature difference between the PV cell and the tank reservoir (${T}_{PV}-{T}_{reservoir}$), as shown in Fig. 5(a), which creates a buoyancy force driving the coolant around the loop. This temperature difference affects both density and pressure difference ($\Delta P$), as expressed using the Boussinesq approximation in Eqs. (4)–(5) and hydrostatic pressure in Eq. (26). The resulting $\Delta P$ along vertical pipe sections can be described by Eq. (27). Fig. 5(b) plots the difference between total pressure changes (${\Delta P}_{2}-{\Delta P}_{1}$) on right and left sides of the loop, which serves as the driving force for fluid circulation. This pressure differential closely follows the temporal profile of (${T}_{PV}-{T}_{reservoir}$) and aligns well with variations in mass flow rate $(\dot{m})$ observed in Fig. 4(a). Notably, the highest values of temperature difference—particularly between 09:00 and 12:00—coincide with peaks in both ${\Delta P}_{2}-{\Delta P}_{1}$ and $\dot{m}$, highlighting the strong thermohydraulic coupling within the system.

26 $$\frac{\partial P}{\partial y}=-\rho g$$

27 $$\Delta P=-\int \rho gdy=-\int {\rho }_{0}g\left(1-\beta \left(T-{T}_{0}\right)\right)dy$$

In summary, Table 4 presents the variation of key parameters with mesh size. As the mesh is refined from 496,000 to 14,864,000 control volumes, the values of all three parameters ($\dot{m}$, ${T}_{PV}-{T}_{reservoir}$, and ${\Delta P}_{2}-{\Delta P}_{1}$) progressively converge. It shows that after 3,356,000 control volumes, those parameters stabilise and become independent of mesh size. The relative error for day-average of $\dot{m}$, ${T}_{PV}-{T}_{reservoir}$, and ${\Delta P}_{2}-{\Delta P}_{1}$ on the case of 3,356,000 control volumes is only 0.54%, 0.32%, and 1.37% compared to the case 7,136,750 and 14,864,000 control volumes, respectively. This level of convergence validates the adequacy of the 3,356,000 control volume mesh for balancing computational efficiency and numerical accuracy. Therefore, this mesh size is adopted for all subsequent simulations in the study.

**Table 4:** Variation of key parameters with mesh size, for ${d}_{CC}$ = 5 mm and ${d}_{P}$ = 100 mm.

Number of control volume	Day-average value		
	$\dot{m}$(kg/s)	${T}_{PV}-{T}_{reservoir}$(K)	∆P_2_−∆P_1_ (Pa)
496,000	0.0117	3.0579	1.8780
1,334,000	0.0119	3.0114	1.9120
3,356,000	0.0121	3.0792	1.8806
7,136,750	0.0122	2.9801	1.8548
14,864,000	0.0123	2.9895	1.8292

## Results

### Radiation filter in the cooling loops

**Fig. 6:**
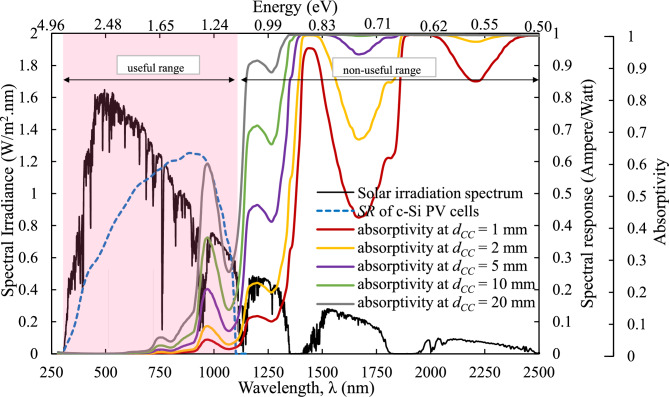
Solar irradiance spectrum at AM 1.5, the spectral response of c-Si PV cells and the absorptivity of pure water for various coolant channel heights, ${d}_{CC}$.

The effectiveness of using pure water as a radiation filter in the NCCL is evaluated. Fig. 6 illustrates the spectral solar irradiance at air mass (AM) 1.5, as specified in ASTM G-173–03, alongside the spectral response ($SR$) of c-Si PV cells and the absorptivity of pure water with varying coolant channel height, ${d}_{CC}$ values as a function of wavelength ($\lambda$). The absorptivity curves are calculated using Lambert’s law of absorption, as detailed in our previous study^24^.

As expected, the absorptivity of pure water increases with the thickness of the cooling channel. A key observation is that most of the solar irradiance absorbed by water lies outside the useful spectral response range of the PV cells (325–1125 nm), particularly in the infrared region beyond 1125 nm. This indicates that pure water can serve as an effective spectral filter, attenuating unwanted infrared radiation that contributes to PV module heating but not electricity generation.

Critically, the graph reveals that at lower channel heights (e.g., ${d}_{CC}$ = 1 mm or 2 mm), water allows most of the useful spectrum to pass through with minimal attenuation, preserving electrical performance while selectively filtering thermal load. As ${d}_{CC}$ increases beyond 5 mm, absorptivity within the non-useful infrared range becomes significant, suggesting an increased capacity to mitigate thermal input. However, further increases to 10 mm and 20 mm begin to show marginal absorptivity even within the lower end of the useful range (near 1000 nm), posing potential trade-offs between thermal control and photon availability for electricity generation.

This result underscores a design optimisation opportunity: coolant channel heights must be carefully selected to balance thermal management and electrical efficiency. Over-filtration risks reducing useful photon flux, while under-filtration fails to sufficiently suppress thermal stress.

### 2-D and 3-D modelling

This section evaluates the impact of different cooling channel height, ${d}_{CC}$, on the NCCL system’s performance, comparing 2-D and 3-D models with tube spacing, ${d}_{P}$ = 100 mm, under the same reference solar irradiance (${G}_{ref}$) conditions. The 2-D simulation results are benchmarked against previous validated studies^24^, while the 3-D model allows a more realistic spatial representation of flow and heat transfer.

**Fig. 7:**
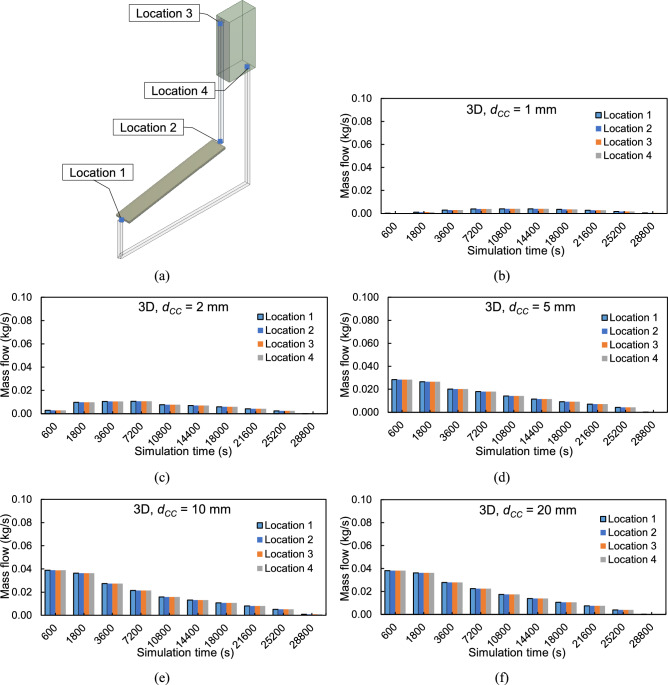
Mass flow rate inside the NCCL system under reference radiation, ${G}_{ref}$ at (**a**) the four different monitoring locations for tube spacing, ${d}_{P}$ of 100 mm and cooling channel heights, ${d}_{CC}$ of (**b**) 1 mm, (**c**) 2 mm, (**d**) 5 mm, (**e**) 10 mm, and (**f**) 20 mm.

Figure 7(a) shows the five monitoring locations within the 3-D NCCL model: the inlet and outlet of the radiation filter box above the PV module and the inlet and outlet of the top tank reservoir. Fig. 7(b-f) display mass flow rates at these locations for various values of cooling channel height (${d}_{CC}$ = 1, 2, 5, 10, and 20 mm). The plots reveal that at each cooling channel height, the mass flow rate is nearly identical across all four monitoring points at every simulation step. This negligible spatial variation validates that the model satisfies mass conservation and indicates a fully converged numerical solution.

**Fig. 8:**
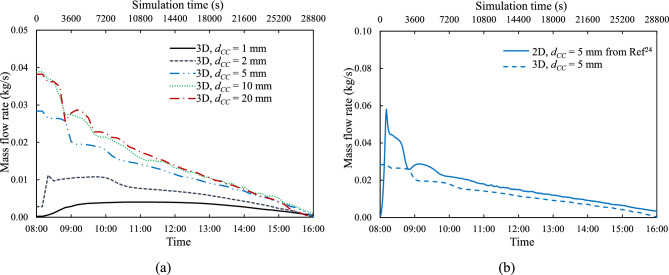
Mass flow rate: (**a**) Time history for various coolant channel heights, ${d}_{CC}$, and (**b**) Comparison between 2-D from Ref^24^ and 3-D models for ${d}_{CC}$ = 5 mm and tube spacing, ${d}_{P}$ of 100 mm.

Fig. 8(a) shows the time history of buoyancy-driven cooling flow $\dot{m}$ for all cooling channel heights at a tube spacing, ${d}_{P}$ of 100 mm. The cooling channel height of ${d}_{CC}$ = 20 mm yields the highest mass flow rate, which is in accord with the corresponding 2-D findings^24^. Also, in agreement with previous findings^24^, Fig. 8(a) shows that the two narrower channels, ${d}_{CC}$ ≤ 2 mm, have a different temporal development of flow rate than the three wider ones. For channel heights, ${d}_{CC}$ greater than 5 mm, the coolant flow rate increases very little with a further increase in channel height. Figure 8(b) compares instantaneous mass flow rate, $\dot{m}$, between 2-D from earlier findings^24^ and 3-D models for ${d}_{CC}$ = 5 mm, revealing that the 2-D model consistently exhibits a higher flow rate due to its design, which resembles a 3-D model with continuous, unseparated connecting tubes (${d}_{P}$ = 0 mm).

**Fig. 9:**
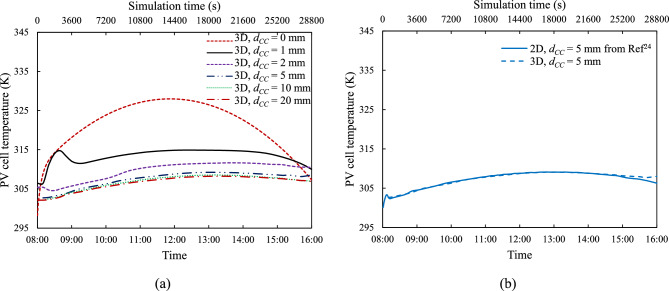
PV cell temperature: (**a**) Time history for different coolant channel heights, ${d}_{CC}$, at a tube spacing, ${d}_{P}$ = 100 mm, and (**b**) Comparison between 2-D from Ref^24^ and 3-D model predictions for ${d}_{CC}$ = 5 mm.

**Fig. 10:**
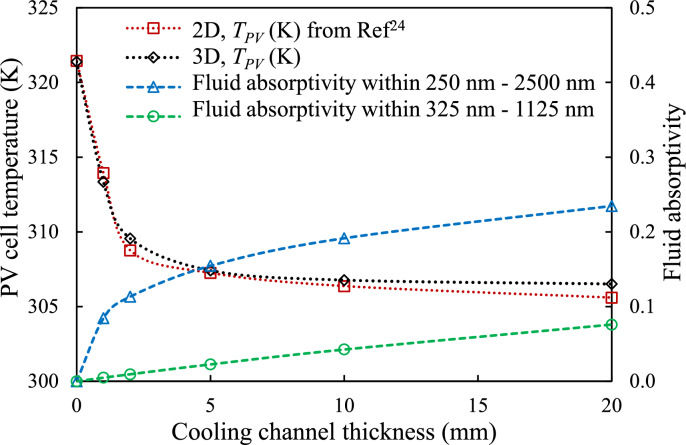
Variation of day-average temperature of PV cell, ${T}_{PV}$ and fluid absorptivity with coolant channel heights, ${d}_{CC}$ at tube spacing, ${d}_{P}$ = 100 mm.

Figure 9(a) shows the time history of the average PV cell temperature (${T}_{PV}$), indicating that higher $\dot{m}$ results in lower temperatures, a trend also demonstrated in our recent 2-D study^24^. For the case where ${d}_{CC}$ = 0 mm, representing a standard FPV panel (without NCCL), the daily average ${T}_{PV}$ is 321.39 K. The daily average ${T}_{PV}$ decreases as ${d}_{CC}$ increases, with values ranging from 313.41 K to 304.90 K for cooling channel heights from 1 to 20 mm, respectively as presented in Fig. 10. Thus, incorporating the NCCL with a radiation filter can reduce the daily ${T}_{PV}$ by up to 15 K, aligning with previous findings^24^. The temperature comparison between 2-D and 3-D models in Fig. 9(b) reveals minimal temperature differences, demonstrating that the 3-D model at ${d}_{CC}$ = 5 mm and with ${d}_{P}$ = 100 mm approximates the simplified 2-D model. This suggests that at this combination of channel height and tube spacing, three-dimensional flow features have a relatively small influence.

Fig. 10 presents a comparison of the day-average ${T}_{PV}$ of 2-D from previous findings^24^ and 3-D models across different coolant channel height, ${d}_{CC}$ values. It shows that, for all ${d}_{CC}$ values, the 3-D model with a tube spacing distance, ${d}_{P}$ = 100 mm is also in close agreement with the simplified 2-D model. Notably, since the 2-D results reported in earlier study^24^ were experimentally validated, this consistency reinforces the reliability of the current 3-D model and supports its extension for more complex geometries and spatial configurations. The close correlation also suggests that the 2-D model, despite its simplifications, remains a valuable tool for preliminary system assessments.

Additionally, Fig. 10 compares ${T}_{PV}$ and fluid absorptivity within a solar radiation filter across different ${d}_{CC}$ values. As discussed in previous findings^24^, fluid absorptivity within the solar radiation filter increases with channel thickness. Pure water has lower absorptivity in the PV spectral response range (325 nm–1125 nm) than in the full solar spectral range (250 nm–2500 nm), indicating its effectiveness as a solar radiation filter to optimise the electrical efficiency of c-Si PV cells.

### Effect of tube spacing

**Fig. 11:**
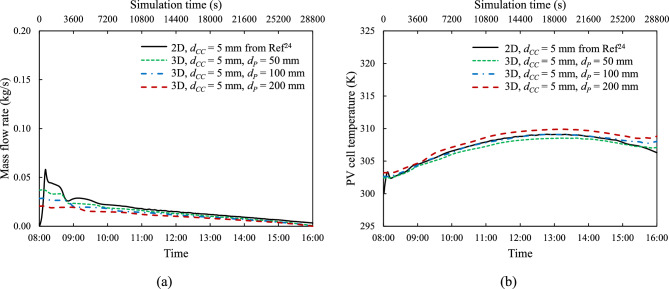
Instantaneous (**a**) coolant mass flow rate, $\dot{m}$ and (**b**) spatially averaged panel surface temperature, ${T}_{PV}$ under reference irradiation, ${G}_{ref}$ with various tube spacings, ${d}_{P}$ and constant coolant channel height, ${d}_{CC}$ = 5 mm.

Fig. 11 compares the time history of the instantaneous mass flow rate $\dot{m}$, and spatially averaged panel surface temperature, ${T}_{PV}$ for both 2-D and 3-D models, all with cooling channel height, ${d}_{CC}$ = 5 mm under the same reference irradiation^24^, ${G}_{ref}$, but with varying tube spacing ${d}_{P}$ values of 50 mm, 100 mm, and 200 mm. As seen in plots of the time-histories of these two parameters presented earlier, for all values of ${d}_{P}$, the mass flow rate is at its highest at the start of the day and then gradually reduces, while the temperature rises gradually to reach a maximum at around 13.00 h and then starts to gradually fall. At the cooling channel height of 5 mm, the effect of the tube spacing length appears to be rather weak.

**Fig. 12:**
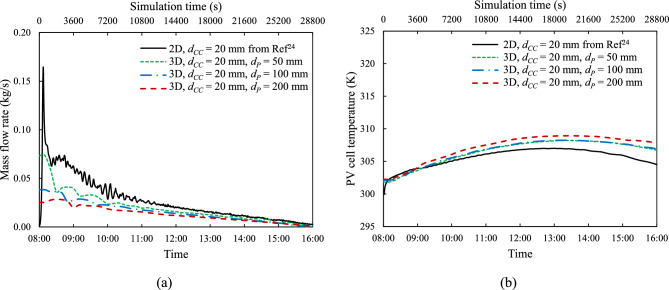
Instantaneous (**a**) coolant mass flow rate, $\dot{m}$ and (**b**) average temperature over PV cell surface, ${T}_{PV}$ under reference irradiation, ${G}_{ref}$ with various tube spacings, ${d}_{P,}$ and constant coolant channel height, ${d}_{CC}$ = 20 mm.

Figure 12 presents similar comparisons for models at a coolant channel height, ${d}_{CC}$ = 20 mm. As far as the mass flow rate plots are concerned the temporal evolution is similar to that at ${d}_{CC}$= 5 mm. For the wider channel, ${d}_{CC}$ = 20 mm, there is also a stronger dependence of the flow rate on the tube spacing, ${d}_{P}$. The corresponding PV temperature comparisons also show similar trends. Additionally, as previously discussed in Figs. 4, 5, the peak in mass flow rate at the beginning of the day corresponds to the maximum values of ${T}_{PV}-{T}_{reservoir}$ and the pressure difference (${\Delta P}_{2}-{\Delta P}_{1}$). The gradual rise and fall of ${T}_{PV}$ throughout the day reflect the polynomial solar irradiance trend described in Eq. (18).

**Fig. 13:**
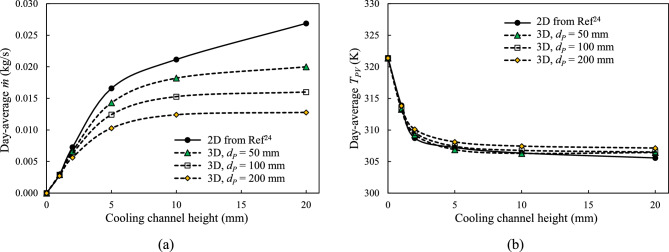
Variation of day average of (**a**) coolant mass flow rate, $\dot{m}$, and (**b**) average temperature over PV cell surface, ${T}_{PV}$ with coolant channel height, ${d}_{CC}$ for different tube spacings, ${d}_{P}$.

Figure 13 summarises the day-average mass flow rate $\dot{m}$ and PV cell temperature ${T}_{PV}$ for all cooling channel heights and pipe spacings. As shown in Fig. 13(a), the day-averaged mass flow rate increases with cooling channel height for both 2-D and 3-D models, with the 2-D model predicting higher values across the range due to its idealised geometry. The 3-D results reveal a strong dependence of $\dot{m}$ on both ${d}_{CC}$ and ${d}_{P}$. In contrast, Fig. 13(b) shows that the ${T}_{PV}$ is primarily governed by the cooling channel height, with minimal sensitivity to tube spacing. The PV panel temperature, ${T}_{PV}$, decreases sharply with increasing ${d}_{CC}$ up to approximately 10 mm, beyond which the reduction plateaus. The ${d}_{CC}$ of 10 mm appears to be the minimum necessary to achieve near-optimal thermal performance, making it a practical design recommendation for efficient integration of NCCL systems in FPV applications.

### Solar irradiance variation

**Fig. 14:**
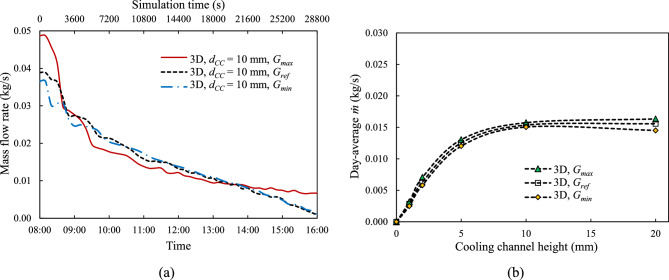
Mass flow rate, $\dot{m}$: (**a**) Time history and (**b**) day average coolant flow rate, $\dot{m}$ under various solar irradiance levels at coolant channel height, ${d}_{CC}$ = 10 mm at tube spacing, ${d}_{P}$ = 100 mm.

**Fig. 15:**
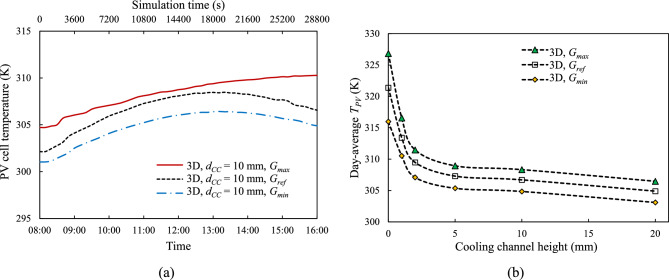
PV cell average surface temperature, *T*_*PV*_: (**a**) Time history and (**b**) Day-average ${T}_{PV}$ under various solar irradiance levels at a coolant channel height, ${d}_{CC}$ = 10 mm and a tube spacing, ${d}_{P}$ = 100 mm.

The effect of solar irradiance on the proposed system has also been studied, to assess its effectiveness at different locations across the Earth and seasons. Three different solar irradiance levels ‒ representing the maximum potential irradiance (${G}_{max}$), minimum possible irradiance (${G}_{min}$), and reference irradiance (${G}_{ref}$) described in "Radiation modelling" ‒ were tested in a 3-D model of the PV cell cooling system, with a cooling channel height, ${d}_{CC}$ = 10 mm, and tube spacing, ${d}_{P}$ = 100 mm. Figure 14 shows the time history and daily average of the coolant flow rate, $\dot{m}$ under these various solar irradiance conditions. Fig. 15 presents the time history and daily average of the PV cell temperature, ${T}_{PV}$ under the same conditions. The effect of solar irradiance on the coolant flow rate appears to be surprisingly weak. As expected, a stronger irradiance results in higher PV cell temperatures.

**Fig. 16:**
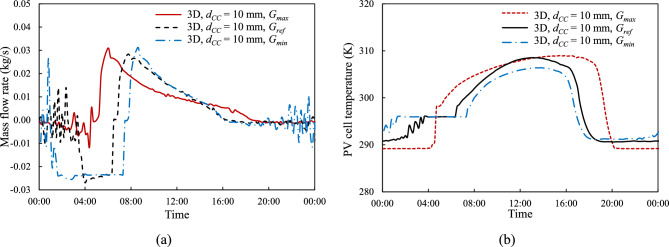
Time history of (**a**) coolant flow rate, $\dot{m}$ and (**b**) PV cell average surface temperature, ${T}_{PV}$ under different solar irradiance over 24 h of simulation at a coolant channel height, ${d}_{CC}$ = 10 mm and tube spacing, ${d}_{P}$ = 100 mm.

To confirm that continuous daily operation is sustainable, Fig. 16 illustrates the performance of the 3-D model with cooling channel height, ${d}_{CC}$ = 10 mm and tube spacing, ${d}_{P}$ = 100 mm, under different irradiance levels over a 24-h simulation cycle. As the different irradiation levels represent the maximum and minimum irradiation across the world—described in "Radiation modelling" and Fig. 3— the start times of the simulation differ, with ${G}_{max}$ beginning at 04:43, ${G}_{ref}$ starting at 06:30, and ${G}_{min}$ starting at 07:28. Additionally, ${G}_{max}$ experiences the longest irradiation duration, while ${G}_{min}$ receives the shortest. However, all irradiation runs are simulated over a 24-h period, with initial conditions consistent across all models, including a mass flow rate of zero and a starting temperature of 298.15 K.

In all cases there is an abrupt rise in coolant flow rate and panel temperature at the start of the day and a similarly abrupt drops at the end of the day. These are consistent with the sharp rises and drops in solar intensity at the start and end of each day, presented in Fig. 3. As previously presented, the time history of mass flow rate in Fig. 16(a) is highest at the beginning of the day, gradually decreasing and approaching zero by the end of the day, in accordance with the low incoming solar irradiation and reduced PV cell temperature. Similarly, Fig. 16(b) shows that the PV cell temperature peaks in the afternoon before decreasing back to lower temperatures.

This 24-h simulation extends the previous 8-h simulation, demonstrating that the cooling loops are capable of returning the PV cell temperature to near its initial condition after a full day’s operation. Furthermore, even under the strongest irradiance, ${G}_{max}$, there is sufficient time overnight for the coolant to return to the morning ambient temperature. At the end of the 24-h simulation, the PV cell temperature is lower than initial condition of 298.15 K. This is attributed to the nighttime ambient temperature being approximately 9 °C lower than its daytime counterpart, with the sky temperature (${T}_{surr}$) being estimated at 20 degrees below ambient temperature—described in "Boundary conditions"—allowing more heat loss overnight, which is consistent with the findings in our earlier study^24^.

**Fig. 17:**
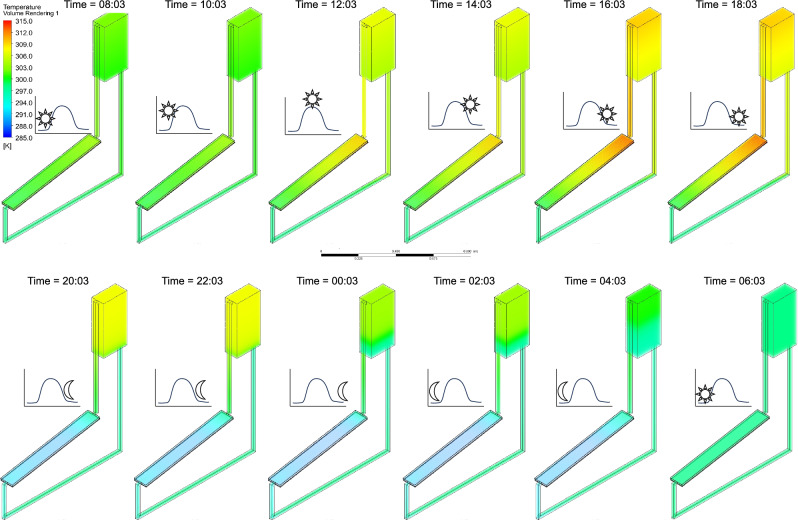
Temperature contours of the NCCL system at different time intervals under the maximum potential solar irradiance (${G}_{max}$) over 24 h of simulation, with a coolant channel height, ${d}_{CC}$ = 10 mm and tube spacing, ${d}_{P}$ = 100 mm.

**Fig. 18:**
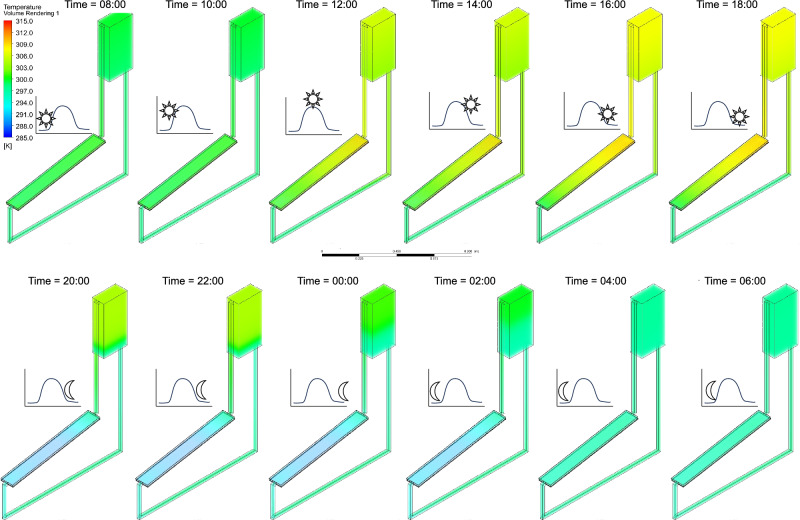
Temperature contours of the NCCL system at different time intervals under the reference solar irradiance (${G}_{ref}$) over 24 h of simulation, with a coolant channel height, ${d}_{CC}$ = 10 mm and tube spacing, ${d}_{P}$ = 100 mm.

**Fig. 19:**
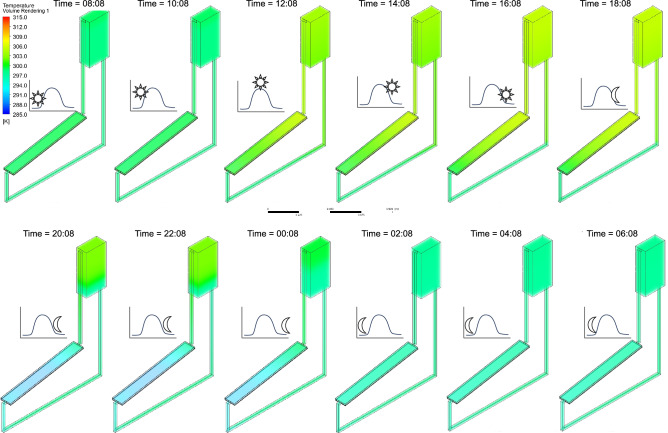
Temperature contours of the NCCL system at different time intervals under the minimum possible solar irradiance (${G}_{min}$) over 24 h of simulation, with a coolant channel height, ${d}_{CC}$ = 10 mm and tube spacing, ${d}_{P}$ = 100 mm.

Additionally, Figs. 17, 18, 19 illustrate the temperature distribution in the NCCL system at twelve-time intervals during the 24-h simulation under ${G}_{max}$, ${G}_{ref}$, and ${G}_{min}$, respectively. Peak temperatures exceed 313 K under ${G}_{max}$, showing strong heat accumulation during midday, while ${G}_{ref}$ and ${G}_{min}$ show progressively lower and more stable profiles.

These results confirm the NCCL system’s ability to dynamically regulate temperature across varying solar conditions. Importantly, temperature uniformity during night hours across all scenarios demonstrates low thermal retention, ensuring operational reset for the next cycle. Supplementary Videos 1, 2, 3 complement these figures by an animated visualisation of these temperature variations throughout the simulation period under {G}_{max}, {G}_{ref}, and {G}_{min}, respectively.

## Discussion

### Analysis of findings

As shown in Figs. 7 and 8, the magnitude of the mass flow rate is strongly affected by the cooling channel height, ${d}_{CC}$. The mass flow rate increases with ${d}_{CC}$, which can be attributed to the fact that the viscous effects are stronger for the narrower channels. The channel height also influences the temporal development of the mass flow rate. For the narrowest channel, ${d}_{CC}$ = 1 mm, due to the strong viscous forces, initially the mass flow rate is very low, it then gradually reaches a maximum at about the middle of the day and then it gradually goes down. As the cooling channel becomes wider, ${d}_{CC}$ = 2 mm, the peak mass flow rate occurs earlier and then eventually, ${d}_{CC}$ = 5 mm and higher, the mass flow rate is highest at the start of the day, when the difference between the PV cell and the reservoir temperatures is greatest, and then it gradually drops. What is also clearly demonstrated in Fig. 8(a), is that initially there is a large increase in mass flow rate with increase in channel height, but beyond 5 mm the rate of increase is considerably reduced and beyond 10 mm the value of the mass flow rate levels off. This suggests that there is not much to be gained by going to channel heights greater than 20 mm.

**Fig. 20:**
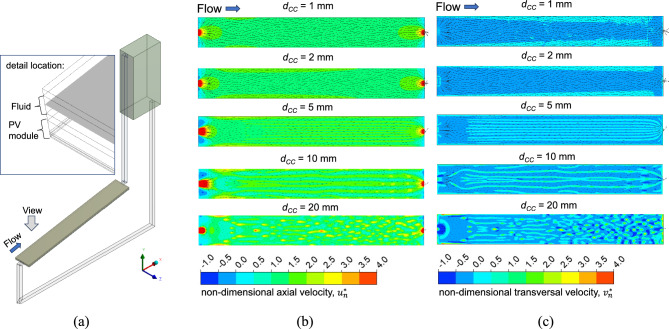
Non-dimensional velocity component analysis: (**a**) Above the PV module, (**b**) axial, ${u}_{n}^{*}$ non-dimensional velocity contours, and (**c**) wall-normal, ${v}_{n}^{*}$ non-dimensional velocity contours for different channel heights, ${d}_{CC}$, at a tube spacing ${d}_{P}$ = 100 mm.

To analyse the buoyancy-generated flow in the cooling channel above the PV module, Fig. 20(a) illustrates the velocity field along the PV module. Equations (28) and (29) define ${u}_{n}$ and ${v}_{n}$ as the axial and transversal velocity component, respectively, while $\theta$ denotes the PV tilt angle ($\theta$ = 10°).

28 $${u}_{n}=u.cos\theta +v.sin\theta$$

29 $${v}_{n}=-u.sin\theta +v.cos\theta$$

The non-dimensional axial velocity (${u}_{n}^{*}$) and non-dimensional transversal velocity (${v}_{n}^{*}$) are defined using Eqs. (30) and (31), where the bulk velocity (${u}_{bulk}$) is calculated using Eq. (32).

30 $${u}_{n}^{*}=\frac{{u}_{n}}{{u}_{bulk}}$$

31 $${v}_{n}^{*}=\frac{{v}_{n}}{{u}_{bulk}}$$

32 $${u}_{bulk}=\frac{\dot{m}}{\rho A}$$

Fig. 20(b, c) display the non-dimensional axial (${u}_{n}^{*}$) and transversal (${v}_{n}^{*}$) instantaneous velocity components of the fluid at noontime ($t=14400 s$). The results confirm that the fluid moves from the bottom to the top due to buoyant forces. A narrower cooling channel results in higher axial velocity because of the reduced cross-sectional area, and the inlet and outlet sections further increase axial velocity due to the narrowing transition from tubes to channels. The wall-normal velocity contours indicate that at the wider cooling channels the flow exhibits three-dimensional features, initially in the form of elongated recirculating cells which eventually break down into more chaotic features. The former have also been observed in the experimental and numerical investigations of buoyancy driven flow in inclined differentially heated cavities by Cooper et al.^38^ and Ammour et al.^39^, respectively.

**Fig. 21:**
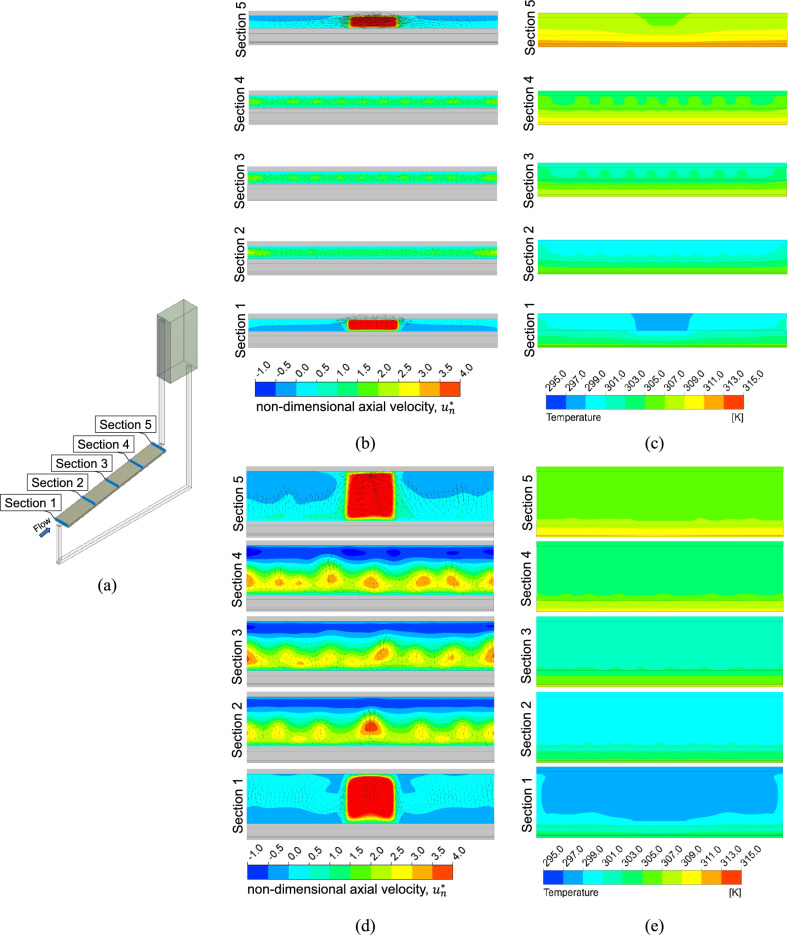
Cross-sectional (**a**) locations along the PV module and channel with tube spacing, ${d}_{P}$ = 100 mm, showing (**b**) contours of the dimensionless axial velocity ${u}_{n}^{*}$ for the coolant channel height, ${d}_{CC}$ = 5 mm, (**c**) temperature contours for ${d}_{CC}$ = 5 mm, (**d**) ${u}_{n}^{*}$ for ${d}_{CC}$ = 20 mm, and (**e**) temperature contours for ${d}_{CC}$ = 20 mm.

 Figure 21(a) shows five cross-sectional locations along the radiation filter box and PV module. These locations include the entrance (section 1, x = 0.00{L}_{PV}), a quarter length in (section 2, x = 0.25{L}_{PV}), the middle (section 3, x = 0.50{L}_{PV}), the third quarter (section 4, x = 0.75{L}_{PV}), and the exit (section 5, x = 1.00{L}_{PV}). Fig. 21(b)–(e) illustrate the instantaneous dimensionless axial velocity, ${u}_{n}^{*}$ and temperature contours across the PV module and cooling channel with ${d}_{P}$ = 100 mm at midday ($t=14400 s$) for ${d}_{CC}$ = 5 mm (Fig. 21(b, c)) and for ${d}_{CC}$ = 20 mm (Fig. 21(d, e)). In the wider channel (${d}_{CC}$ = 20 mm), the symmetric pattern of secondary convective flows with rotating vortices—typical of low Rayleigh number scenarios in channels heated from below^40^—is clearly observed, whereas this effect is less prominent in the narrower channel (${d}_{CC}$ = 5 mm). As also commented earlier, there are several longitudinal vortices along the length of the channel. More significantly, there is a sizeable region of flow reversal along the top surface of the cooling channel, denoted by the blue regions in the velocity contour plots of Fig. 21(d). The latter is a consequence of the unstable thermal stratification, evident in the temperature contour plots of Fig. 21(b, d) and which has only been observed for the wider channel.

The tube spacing (${d}_{P}$) in the 3-D models plays a critical role as it influences the number of connecting tubes (${N}_{P}$) between the tank reservoir and the PV module. Figs. 11 and 12 demonstrate that a) there are only modest differences between the three- and two- dimensional predictions and b) as the number of tubes increases (lower ${d}_{P}$), the mass flow rate temporal profile rises and the that of the temperature falls towards the two-dimensional variations.

Fig. 13 summarises that for the same PV cell width (${W}_{PV}$) of 1 m, the 2-D model consistently has the highest mass flow rate at all channel heights, ${d}_{CC}$. This occurs because the 2-D model is equivalent to a 3-D model with continuous, unseparated connecting tubes, which offers a lower resistance to the flow. Consequently, the 3-D model with the smallest ${d}_{P}$ of 50 mm also exhibits a higher mass flow rate than the larger tube spacings. A higher mass flow rate is crucial for efficiently releasing heat from PV cells, resulting in lower temperatures. Conversely, the 3-D model with ${d}_{P}$ = 200 mm shows the highest cell temperature, ${T}_{PV}$, while the 2-D model has the lowest.

The 2-D model shows a slightly lower ${T}_{PV}$ over the 3-D models, with average ${T}_{PV}$ differences of 0.48 K, 0.59 K, and 0.99 K for ${d}_{P}$ of 50 mm, 100 mm, and 200 mm, respectively. It indicates that reducing ${d}_{P}$ from 100 to 50 mm doesn’t significantly affect the temperature compared to the 2-D model. It suggests that the ${d}_{P}$ = 100 mm is sufficient to optimise the 3-D model in the NCCL system.

The results from the solar irradiance variation study indicate that varying irradiance levels has only a modest impact on the coolant flow rate, which suggests that the natural convection cooling remains effective at all Earth locations, but they significantly impact the ${T}_{PV}$. As expected, ${G}_{max}$, which represents the highest solar irradiance, results in the highest PV cell temperature at all cooling channel heights. On average there is a 2.64 °C difference in PV cell temperature from one location to the next.

An extended simulation from 8 h to a 24-h cycle reveals that by the end of the day, the PV cell temperature, ${T}_{PV}$, and equally importantly the temperature of the coolant, returns close to its initial conditions. This demonstrates that the proposed NCCL system can thermally reset itself after a full day’s operation, ensuring continuous cooling system functionality day after day.

The FPV-NCCL configuration incorporates a fixed panel tilt angle of 10° to facilitate self-cleaning by natural dust removal, based on the minimum angle recommended for PV panels^41,42^. While this angle simplifies maintenance, it departs from conventional practice, where tilt angles are adapted based on latitude to optimise seasonal energy yield. Previous studies have shown that lower tilt angles (< 30°) favour summer performance, while steeper angles (30°–60°) enhance winter energy capture and promote convective cooling due to increased airflow beneath the panel^9^. Despite the conservative setting of the tilt angle, this study demonstrates that effective heat transfer and coolant circulation are maintained, validating the feasibility of the simplified design.

While in the current model parameter such as the NCCL tube diameter (${d}_{NCCL}$), top tank reservoir (${l}_{R}$ and ${h}_{R}$), and material properties are held constant to isolate the effect of the cooling strategy, this introduces a limitation. These variables are known to significantly affect thermal and hydraulic performance and merit further parametric investigation. Future work should consider exploring these factors and their interactions to comprehensively optimise FPV-NCCL system design across diverse climatic and operational contexts.

Study on the environmental effect highlights that FPV can influence aquatic habitats by blocking sunlight, modifying water circulation and surface interactions, and affecting bottom-dwelling organisms and aquatic wildlife^43^. With proper monitoring of water quality, biodiversity, and emissions, FPV impacts can be managed while supporting global decarbonisation efforts^44^. The inclusion of the NCCL system also leads to the possibility of further environmental implications, such as potential leaks, and thermal discharge. As our proposed cooling system is a closed loop one and uses pure water, this impact can be managed. Even under peak operating conditions, the simulated temperature increase remains well below 10 °C, which is within acceptable ecological thresholds should any unintended discharge occur^45,46^.

Long-term operational challenges such as coolant degradation, biofouling, and material weathering from prolonged exposure to UV radiation and thermal cycling also warrant consideration. These effects can impair system performance and reduce lifespan if left unaddressed. The NCCL system, by maintaining lower module temperatures, inherently reduces thermal ageing. Additional measures such as antifouling coatings, self-cleaning surfaces, and wave-assisted rinsing can limit biological accumulation and surface contamination^47^. Moreover, modular design and routine monitoring are essential to ensure maintainability and long-term durability of FPV-NCCL installations in aquatic environments.

Furthermore, the cost–benefit analysis of the proposed NCCL-enhanced FPV system has been previously carried out in our earlier study^16^, which remains applicable here, given the similarity in design parameters. The analysis compared the levelised cost of electricity (LCOE) and payback period (PP) for conventional FPV panels and FPV panels integrated with the NCCL system. Using benchmark installation costs provided by the National Renewable Energy Laboratory (NREL), the study revealed that while the addition of the NCCL system entails a modest increase in initial capital expenditure, the resulting improvement in electrical efficiency significantly offsets this cost. Specifically, the LCOE was reduced by up to 10.19%, and the PP shortened by up to 11.90%, relative to standard FPV systems. These findings highlight the economic viability of integrating NCCL technology into FPV applications, offering an attractive return on investment for stakeholders while enhancing long-term energy performance^16^.

### Performance comparison

Table 5 compares the findings of this study with those of previous research on radiation filters and cooling systems in PV panels. Most hybrid radiation filter and cooling systems are designed for photovoltaic-thermal (PV/T) collectors, which are evaluated based on two key performance metrics—electrical efficiency and thermal efficiency. Electrical efficiency measures the percentage of incident solar energy converted into electricity by the PV module, while thermal efficiency quantifies the percentage of incident solar energy converted into useful thermal energy collected by the cooling system.

**Table 5:** Comparison of results for radiation filters in various PV panel studies.

Authors	Method and application	Results
Crisostomo et al.^25^	Investigated radiation filters for hybrid photovoltaic-thermal (PV/T) collectors, employing pure water and Ag-SiO_2_ nanofluid with a constant thickness of 12 mm in a forced convection system	Pure water and Ag-SiO_2_ nanofluid (2.5 wt% concentration) reduced electrical efficiency by 33% and 51%, respectively, due to absorption in the useful solar wavelength range. Thermal efficiency improved by 4% and 12%, respectively. However, additional pumping power is required at a flow rate of 1 kg/min
Han et al.^48^	Studied radiation filters for PV/T system using stationary deionised water and Ag-water nanofluid with a constant thickness of 10 mm in a forced convection system	Deionised water and 5.3 ppm Ag-water nanofluid reduced electrical efficiency by 10% and 23%, respectively, while they contributed to thermal efficiency by 14% and 22%. Despite the improvements, the system requires additional pumping power to circulate the coolant
Huaxu et al.^49^	Studied radiation filters for concentrating PV/T system using glycol-ZnO nanofluid at varying concentrations (11.2, 22.3, 44.6, and 89.2 ppm) in a forced convection system	Increasing the ZnO concentration from 11.2 ppm to 89.2 ppm resulted in a reduction in electrical efficiency by 11.3% up to 19.8%, attributed to the lower transmittance, while the thermal efficiency improved ranging from 7.4% to 10.97%. However, additional pumping power is needed to circulate the coolant
Abdelrazik et al.^50^	Investigated radiation filters for PV/T panel using 0.0005 wt% Ag-water nanofluid with a variable thickness of 2–20 mm in a forced convection system	Increasing the channel thickness enhanced the coolant’s light absorption but reduced electrical efficiency by 12.7%. Higher coolant flow rates (3.6–36 L/hour), enabled by pumping power, significantly lowered ${T}_{PV}$, boosting thermal efficiency by 50.8%, although electrical efficiency improved marginally by 5.6%
Present study	Proposed a hybrid radiation filter and NCCL for FPV panels, using pure water with a variable thickness of 1–20 mm in a natural convection system	Proposed system reduces daily ${T}_{PV}$ by up to 15 K, increases electrical efficiency by 3% (by controlling the coolant thickness), and contributes to thermal convective cooling —analogous to thermal efficiency in PV/T systems—by up to 64.48%, all achieved without requiring pumping power

Since the PV cell average temperature, ${T}_{PV}$, results in this study show only minor differences compared to the 2-D model, the electrical and thermal efficiency values are referenced from our previous study^24^. These comparisons highlight that the proposed cooling system offers excellent radiation filtering and cooling performance, with the added advantage of filtering out non-useful wavelengths, and all without the need for pumping power.

In addition, Table 6 provides a comparative assessment of the proposed NCCL system against other passive cooling strategies that may be applicable to FPV panel. Since all evaluated systems operate without the need for pumping power, the comparison is primarily based on their ability to reduce PV cell temperature and enhance electrical performance. The proposed NCCL system achieves a daily reduction in PV cell temperature (${T}_{PV}$) of up to 15 K, outperforming many alternative methods. Although the improvement in electrical efficiency is relatively modest (approximately 3%), due to partial attenuation of the solar irradiation, this trade-off is justified by the dual-functionality of the NCCL system. Unlike conventional passive systems, the NCCL configuration uniquely combines spectral irradiation filtering and buoyancy-driven convective cooling. Notably, the system contributes up to 64.48% of the thermal convective cooling through natural convection, a thermal performance that is rarely matched by other passive techniques.

**Table 6:** Benchmarking passive cooling strategies suitable for FPV applications.

Authors	Cooling method	Results
Chan-Dzib et al.^51^	Aluminium fins with varying geometries—rectangular and curled—were mounted on the rear surface of PV modules. The designs varied in fin length, width, curl degree, perforation and convectional area	Experimental results showed that curled aluminium fins reduced panel temperatures by up to 8 °C, resulting in a 3–4% increase in electrical output. Rectangular fins yielded a more modest improvement, enhancing energy output by 2–2.9% compared to an uncooled PV system
Ma et al.^52^	Copper heat pipes (15 mm × 1.5 mm cross-section) were integrated onto the rear surface of the PV module. The heat pipes consisted of a 400 mm evaporation section, a 150 mm condensation section, and a 50 mm adiabatic section, using pure water as the working fluid	The heat pipe (HP) system reduced the PV cell temperature from 52.79 °C to 45.32 °C (a 10.36% reduction) and improved electrical power output by 9.13% compared to an uncooled PV module. Further optimisation studies revealed that the system performs best when equipped with 14 heat pipes and a 30° inclination angle for the condensation section, under which the PV-HP system achieves its maximum power output
Hachem et al.^53^	Phase change material (PCM) cooling using petroleum jelly was applied at the rear of the PV panel. Composite PCMs were also formulated by adding copper and graphite powders at mass ratios of 20% and 10%, respectively, to the base petroleum jelly (70%)	Experimental testing in Al-Khyara, Lebanon, demonstrated that pure petroleum jelly PCM reduced PV panel temperatures by up to 6.5 °C (with an average reduction of 2.7 °C) and improved electrical efficiency by approximately 3.0%. The composite PCM achieved even greater thermal performance, lowering panel temperatures by up to 6.3 °C (average 5.6 °C) and increasing efficiency by 5.8%
Hasan et al.^54^	Paraffin-based PCM (10.2 L) was enclosed in rear-mounted containers on the PV panel and solidified to allow phase-change cooling, with a 7 cm headspace to accommodate thermal expansion	A one-year field study in Al Ain, UAE, showed seasonal variability, with temperature reductions of up to 13 °C in April and 8 °C in June, averaging 10.5 °C annually. The system increased annual PV power output by 5.9%, highlighting the effectiveness of PCM with an optimised melting point for passive cooling in hot climates
Li et al.^55^	Hydrogel-based atmospheric water harvesting (AWH) material composed of carbon nanotube (CNT)-embedded cross-linked polyacrylamide (PAM) and calcium chloride (PAM–CNT–CaCl₂) for sorption–desorption cycling	Experimental testing in outdoor conditions in Saudi Arabia demonstrated that the system reduced PV module temperatures by at least 10 °C under 1.0 kW/m^2^ solar irradiance. The system delivered up to 295 W/m^2^ of cooling power and enhanced electrical output by 13–19%

Furthermore, by selectively filtering non-useful solar irradiation wavelengths—which contribute to thermal load but not electricity generation—the NCCL system not only reduces ${T}_{PV}$ but also protects the PV cells from thermal degradation and operational stress. This integrated approach enhances both system reliability and long-term performance, making it a promising and scalable solution for FPV applications.

## Conclusions

This study has employed a multi-physics numerical model to examine a hybrid system combining a radiation filter with a natural convection cooling loop for a floating photovoltaic system. The innovative 2-D and 3-D models were then used to optimise the cooling loop’s geometry and to assess the system’s performance under various global solar irradiance patterns. The results consistently show that increasing the coolant channel height in both 2-D and 3-D models enhances the buoyancy-driven coolant flow rate, which lowers the operating temperatures. The 2-D model, which corresponds to the limiting case of a 3-D model with continuous, unseparated connecting tubes, exhibits a higher coolant mass flow rate than the 3-D model. However, with a tube spacing of 100 mm between connecting tubes, the 3-D model’s photovoltaic cell temperature closely matches that of the 2-D model, with only a 0.16% difference. Testing this model under different levels of solar irradiance, – ranging from maximum to minimum, – demonstrated that varying irradiance have a weak impact on the coolant mass flow rate, although stronger irradiance results in a higher temperature distribution. Notably, even at the minimum irradiance level, the proposed system effectively generates fluid flow and reduces photovoltaic temperature. The 24-h simulations further confirmed that the cooling loops can consistently maintain optimal photovoltaic cell temperatures under any solar irradiation conditions throughout daily operations.

## Supplementary Information

Supplementary Video 1.

Supplementary Video 2.

Supplementary Video 3.

## Data Availability

The datasets generated during and/or analysed during the current study are available from the corresponding author on reasonable request.
